# Comparison of Autologous Rectus Fascia and Synthetic Sling Methods of Transobturator Mid-Urethral Sling in Urinary Stress Incontinence

**DOI:** 10.7759/cureus.23278

**Published:** 2022-03-17

**Authors:** Serkan Dogan

**Affiliations:** 1 Urology, Sancaktepe Training and Research Hospital, Istanbul, TUR

**Keywords:** urology, urinary stress incontinence, tot, synthetic material, autologous fascia

## Abstract

Objective: This study aims to compare the efficacy and complications between mesh obtained from the autologous rectus fascia and synthetic mesh used in transobturator tape procedure in the surgical treatment of urinary stress incontinence.

Methods: A total of 62 female patients who underwent operation due to urinary stress incontinence were included in the study. From these, 31 patients underwent autologous rectus fascia with mid-urethral sling (Group 1), and the remaining 31 patients underwent the same operation using synthetic multilaminar propylene sling (Group 2). The groups were compared preoperative and postoperative according to results of Urogenital Distress Inventory-Short Form (UDI-6) and Incontinence Impact Questionnaire-Short Form (IIQ-7). Demographic characteristics, surgical features, and complications were also compared between the groups. P < 0.05 values were considered statistically significant.

Results: The mean age was found as 54.74 ± 0.87 in Group 1 and 55.58 ± 0.76 in Group 2. There was no significant difference between the groups in terms of the preoperative and postoperative UDI-6 results ​​(p=0.258, p=0.349). Similarly, the preoperative and postoperative IIQ-7 results did not show a significant difference between the groups (p=0,483, p=0,367). There was also no significant difference in demographic characteristics and complications between the groups. Only the mean operational time was significantly longer in Group 1 (p=0.029).

Conclusion: Transobturator tape procedure with autologous rectus fascia is as effective and safe as synthetic mesh. This procedure provides an inexpensive and consumable option without posing a risk of mesh erosion and with low complication rates.

## Introduction

Urinary incontinence is an objectively detectable urine leak. It has attracted attention as a social and hygienic problem by the International Continence Association (ICS) [1]. There are three types of incontinence: stress, urge and mix. Among these types, stress urinary incontinence (SUI) is defined as leakage of urine when intravesical pressure exceeds urethra pressure, without an increase in detrusor activity [2]. SUI is a significant problem that affects both health and social life, especially in women. SUI globally affects over one out of every four women between ages 30 and 60 and constitutes approximately 77% of all incontinence cases [3]. Many surgical techniques have been suggested in the treatment of this large-scale problem. Urethra plication, retropubic fixation of periurethral tissues, colposuspension of the bladder neck, periurethral injections, and sling operations are the most preferred surgical treatments. The selection of the initial procedure is important, as it generally has the highest surgical success rate [4-6]. Von Giordano first described suburethral sling operations in 1907, the widespread use of the procedure in clinical practice did not occur until the late 1970s. New procedures have been implemented with the development of synthetic suspension materials after the 1990s in the sling operations in which autologous rectus fascia (ARF) or fascia lata were previously used [7]. Among these, transvaginal tape (TVT) and transobturator tape (TOT) procedures come forward as the most preferred sling operations [5]. TOT operation is a mid-urethral sling operation described for the first time by De Lorme in 2001 [8]. Since then, many studies have been published, suggesting that TOT has fewer complications than TVT [9,10]. The other treatment methods include stem cell injection, platelet-rich plasma (PRP), and laser ablation [11]. However, the number of studies comparing these materials is scant. Although synthetic materials have been used widely recently, some side effects, especially erosion and dyspareunia, cause a discussion of the safety of these materials. In this study, we aimed to compare ARF and synthetic sling material used in TOT surgery in terms of effectiveness and complications.

## Materials and methods

A total of 62 female patients who underwent an operation for SUI between November 2014 and December 2017 were included in the study. While 31 patients were applied mid-urethral sling using ARF (Group 1), the other 31 patients were applied the same procedure using synthetic multilaminar propylene sling material (TVT Obturator System - Ethicon-Gynecare, OH, USA) (Group 2). TOT procedures were performed by a single surgeon (SD). Patients with a history of pelvic operation, previous incontinence surgery, pelvic radiotherapy, uncontrolled diabetes mellitus (DM), neurological disease, prominent mixed incontinence, and cystocele above grade 2 following pelvic organ prolapses measurement were excluded from the study. Systematic randomization was performed for the grouping of the patients. Patients meeting the inclusion criteria were randomly assigned to two groups according to their order of admission. 

Demographic information of the patients (age, number of births, menopause status, BMI, follow-up duration) as well as preoperative and postoperative hemoglobin (Hgb) levels, and quality of life scores measured by the results of Turkish-translated Urogenital Distress Inventory-Short form (UDI-6) and Incontinence Impact Questionnaire-Short form (IIQ-7) were compared. Complete urine analysis, urine culture, urinary system ultrasonography, and tests were performed in both the preoperative and postoperative periods. Patients underwent preoperative and postoperative cough stress tests (MMK: Marshall-Marchetti-Krantz) [12]. The two groups were also compared according to operational time and complications. While postoperative Hgb was measured 24 hours after the operation, quality of life was assessed three months after the surgery. Complications that occurred within the first 15 days following the operation were considered early complications, while those that occurred after this period were deemed to be late complications.

**Figure 1:**
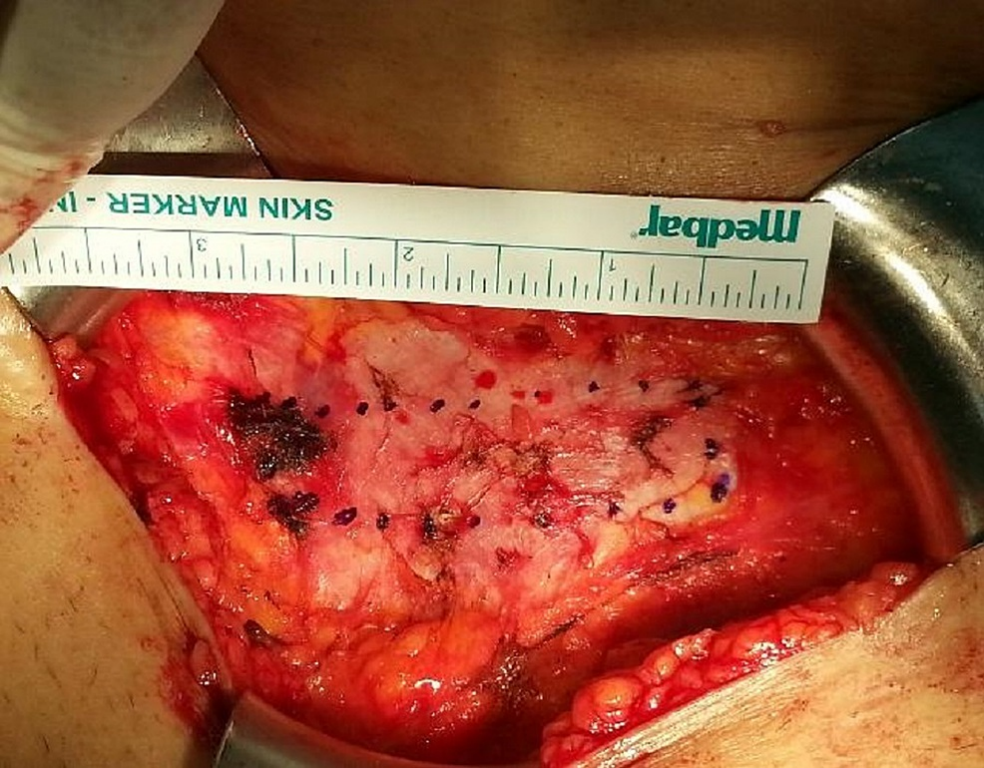
Pfannenstiel was applied over 3 cm above the pubis. Subcutaneous tissue was then dissected with monopolar electrocautery. The circumference of the rectus fascia was enlarged by blunt dissection. An 8 cm x 1.5 cm section of the fascia marked by a pen of surgery.

**Figure 2:**
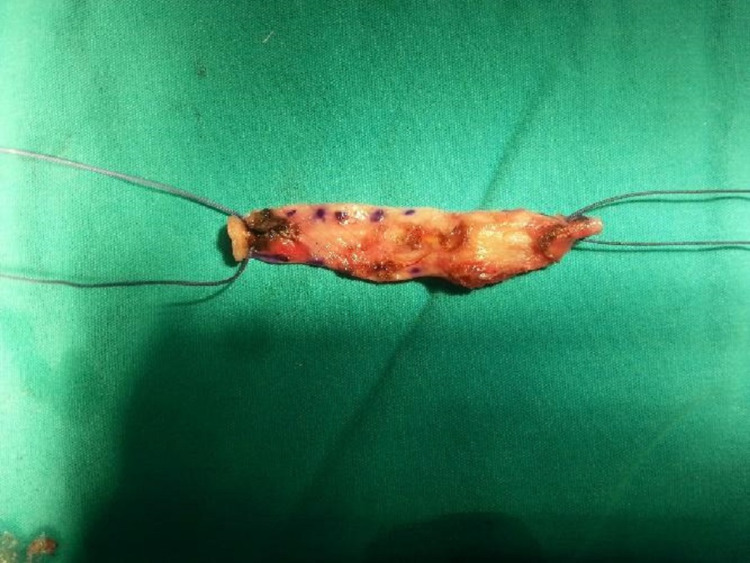
Rectus fascia was resected and lateral attachments were created on the short edges of the fascia with 1-0 vicryl.

Patients of both groups were operated on in a lithotomy position. In Group 1 patients, rectus fascia was first approached with Pfannenstiel incision from 3 cm above the pubis. After the skin incision, the subcutaneous tissue was opened with a proper dissection by monopolar electrocautery and the rectus fascia was reached. Fascia circumference was enlarged by blunt dissection. An 8 cm x 1.5 cm section of the fascia marked by a pen of surgery was resected, and layers were closed according to the patient's anatomy. The skin was subcutaneously sutured. Lateral attachments were created on the short edges of the fascia with 1-0 vicryl (Figures 1, 2).

**Figure 3:**
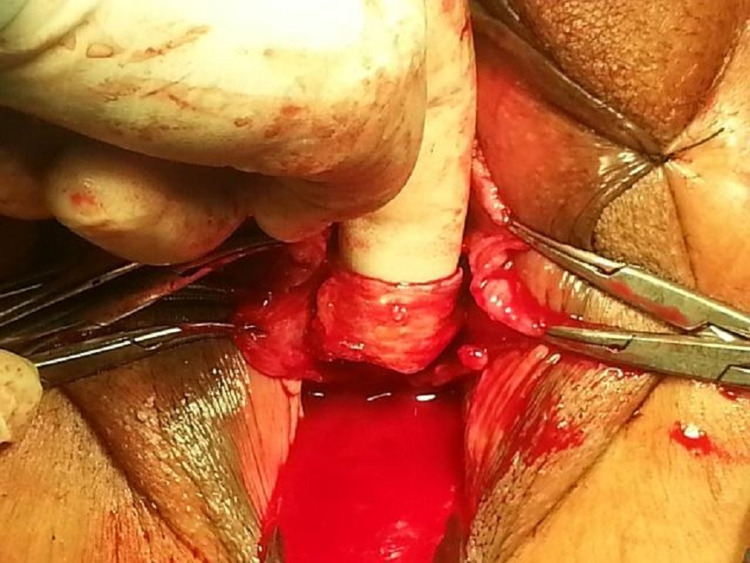
A midline anterior vaginal wall incision is made and periurethral dissection is carried to the retropubic space. TOT needle passer is inserted into the retropubic space and directed paraurethrally under finger guidance (by the De Lorme method). The sling material was attached to the needle tip and placed under the mid urethra.

**Figure 4:**
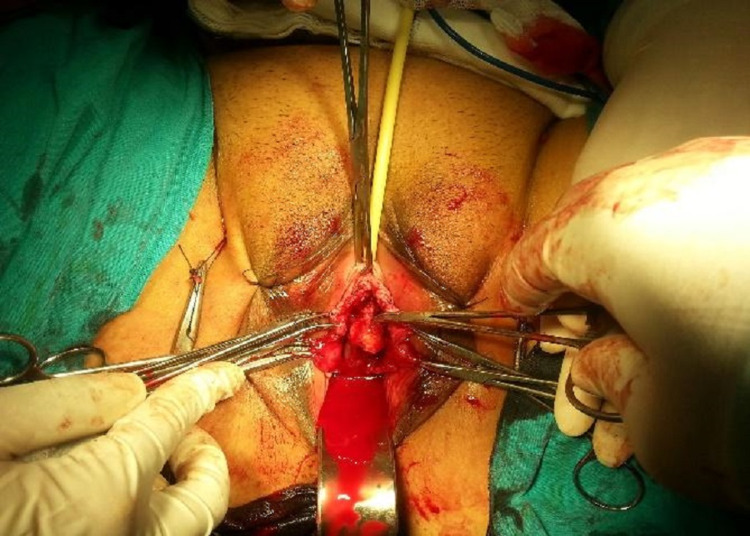
TOT needles were removed and the sling material tension was adjusted.

In Group 2, mid-urethral sling was applied transvaginally with the DeLorme method. TOT needle passer is inserted into the retropubic space and directed paraurethrally under finger guidance. The sling material tension was adjusted (Figures 3, 4).

Vaginal povidone-iodine tampon was inserted and the urethral catheter was removed one day after the operation. Patients were recommended 6-8 weeks of sexual abstinence and discharged. Patients were invited for routine follow-up one, two, six weeks, and three months after the operation. Physically examination was performed at each control. The cough stress test was conducted in the 3rd month, and UDI-6, and IIQ-7 questionnaires were applied.

Ethics considerations

The study was approved by the Ethics Committee of Health Sciences University, Turkiye Yuksek Ihtisas Training and Research Hospital in January 2018 with the decision number 3715. The study was prepared in accordance with the ethical principles of the Declaration of Helsinki. Informed consent forms were obtained from all patients.

Statistical analysis

Data obtained in this study were statistically analyzed using SPSS (Statistical Package for Social Sciences for Windows version 22.0, IBM Inc., Chicago, IL, USA). Normality of the data was tested using the Kolmogorov-Smirnov method. Normal distribution was observed according to demographic characteristics of age, body mass index (BMI), operation length, and also preoperative and postoperative Hgb of the groups. These parameters were then compared with the Student's t-test. Menopause status, cystocele presence, and cough stress test results were compared to the Chi-Square test since they were nominal variables. The number of births and the follow-up period did not show the normal distribution and were compared with the Mann-Whitney U test. Preoperative and postoperative IIQ-7 and UDI-6 scores were compared with paired-samples T-test among themselves, and the efficacy of these evaluations was compared between the groups with Student's t-test. A comparison of the Hgb difference between the groups was also assessed with the Student's t-test. P<0.05 values were considered statistically significant.

## Results

Preoperative and postoperative IIQ-7 and UDI-6 scores were compared within each group, significant differences were revealed (Table 1).

**Table 1:** Comparison of IIQ-7 and UDI-6 scores between study groups IIQ-7: Incontinence Impact Questionnaire–Short Form, UDI-6: Urogenital Distress Inventory–Short Form

	Preop	Postop	P-value
Group 1 IIQ-7	9.51±0.45	1.83±0.39	<0.001
Group 1 UDI-6	9.86±0.56	3.07±0.49	<0.001
Group 2 IIQ-7	9.33±0.66	1.74±0.48	<0.001
Group 2 UDI-6	9.54±0.69	2.92±0.44	<0.001

Group 1 preop and postop IIQ-7 averages were found as 9.51 ± 0.45 and 1.83 ± 0.39, respectively (p=0.001). Group 2 preop and postop IIQ-7 averages were 9.33 ± 0.66 and 1.74 ± 0.48, respectively (p=0.001). Group 1 preop and postop UDI-6 averages were found as 9.86 ± 0.56 and 3.07 ± 0.49, respectively (p=0.001). Group 2 preop and postop UDI-6 averages were found as 9.54 ± 0.69 vs 2.92 ± 0.44, respectively (p=0.001). When the two groups were compared statistically, there was no significant difference between preoperative and postoperative IIQ-7 values ​​(p=0,483, p=0,367). Similarly, when the two groups were compared, there was no significant difference between both preoperative and postoperative UDI-6 values ​​(p=0.258, p=0.349). No statistically significant difference was found when differences in preoperative and postoperative IIQ-7 scores and differences in preoperative and postoperative UDI-6 scores were compared according to groups (p=0.494, p=0.06, respectively). Furthermore, there was no significant difference between the groups according to a difference in preoperative and postoperative Hgb levels (p=0.244) (Table 2).

**Table 2:** Score changes and Hgb changes between study groups IIQ-7: Incontinence Impact Questionnaire–Short Form, UDI-6: Urogenital Distress Inventory–Short Form, Hgb: Hemoglobin

	Group 1	Group 2	p value
IIQ-7 difference	7.68±0.55	7.59±0.84	0.50
UDI-6 difference	6.79±0.86	6.62±0.88	0.06
Hgb difference	0.76±0.10	0.69±0.11	0.24

There was no statistically significant difference between the demographic characteristics of the groups. There was no significant difference between age, BMI, menopause status, cystocele presence, number of births, and preoperative Hgb levels. While there was no significant difference in follow-up periods and postoperative Hgb levels between the groups, operation length was significantly lower in Group 2 (p=0.029) (Table 3).

**Table 3:** Demographic characteristics and surgical data of the study groups BMI: Body mass index, Hgb: Hemoglobin

	Group 1	Group 2	p value
Age (years)	54.74 ±0.87	55.58±0.76	0.41
BMI (kg/cm^2^)	30.28±0.55	29.55±0.44	0.14
Number of births	2.54±0.16	2.70±0.17	0.60
Preop Hgb (mg/dl)	11.97±0.10	12.09±0.11	0.78
Postop Hgb (mg/dl)	11.21±0.09	11.40±0.11	0.26
Length (min)	56.61±1.39	41.29±0.76	0.03
Follow-up (months)	9.9±0.89	10.4±0.98	0.78

Twenty-four (77%) patients in Group 1, 25 (81%) patients in Group 2 were in menopause. Cystocele was present in 12 (39%) patients in Group 1 and 14 (45%) patients in Group 2. There was no difference between menopause status and cystocele presence between the groups (p=0.775, p=0.607, respectively).

In the preoperative cough stress test, 29 (94%) patients in Group 1 and 27 (87%) patients in Group 2 were positive. In the analysis performed at the postop 12th week, two (6%) patients in Group 1 and three (10%) patients in Group 2 were positive. There was no significant difference in preop and postop cough stress test results between the groups (p=0.390, p=0.641, respectively). The complications and Clavien classification of the groups are presented in Table 4.

**Table 4:** Complications of the study groups

Complications	Group 1 (n:31)	Group 2 (n:31)	Clavien Classification
Hemorrhage	1 (3.1%)	0	3b
Urinary Retention	2 (6.2%)	3 (9.4%)	3a
Hematoma	1 (3.1%)	1 (3.1%)	2
Mesh Erosion	0	1 (3.1%)	3b
Wound infection	2 (6.2%)	0	2
Urinary infection	2 (6.2%)	3 (9.4%)	2
Urethra injury	0	1 (3.1%)	3b
Bladder injury	1 (3.1%)	0	3b
Late-period pain	0	2 (6.2%)	2
Dyspareunia	1 (3.1%)	2 (6.2%)	2

## Discussion

SUI is an increasingly common pathology that negatively affects social life and quality of life in women. The surgical treatment of this pathology is performed more and more frequently [13]. There are different methods in surgical treatment among which the middle urethra sling procedure is the most commonly used. Along with autologous fascias, synthetic materials are preferred more frequently as suspension material. In surgical procedures over recent years, the TOT procedure is the foremost in prominent mid-urethral sling operations [14]. The success rate of TOT surgery is very high. Although complication rates (especially organ injuries) are relatively less than other methods, the most critical problems are caused by sling material [15].

In our study, we found that ARF and synthetic mesh had similar efficacy in TOT operations. However, the ARF group had specific complications due to fascia resection, and the synthetic mesh group due to foreign objects. We evaluated both methods with generally similar reliability margins. The selection of method is determined by patient preference, surgeon experience, mesh availability, and conditions that limit the use of mesh or rectus fascia. While choosing the material, patient-specific features that may have adverse effects on wound healing, including previous pelvic surgery, suprapubic incisions, DM, metabolic syndrome, obesity, may direct doctors to use synthetic materials. However, the choice of the autologous fascia may be preferred in patients who have previously had problems with mesh used in different surgeries and in situations that may increase the risk of mesh erosion, such as vaginal atrophy. We did not include patients with previous pelvic surgeries, patients with malignancy, and patients with uncontrolled DM to obtain homogeneous groups. There is a need for more publications on this subject with larger patient groups.

There are relatively few studies in the literature that compare the efficacy and morbidity of ARF and synthetic sling material in transobturator surgery of SUI. We performed TOT as a surgical procedure because it is a fast and reliable method. Although the follow-up period has not yet progressed very long, both groups showed significant recovery. While we did not see many complications, fascia excision in the ARF group is a disadvantage. However, mesh erosion only developed in one patient, while sling-related pain occurred in two patients, both in the synthetic mesh group. Aside from these, there was no noticeable difference in perioperative complications.

Linder and Elliot performed transobturator sling operation with ARF in 33 patients and presented the short-term outcomes. While they concluded that this method is promising, further study is needed with a broader series [16]. Al-Azzawi studied and compared the results of 80 patients who underwent TOT operation in which 40 patients applied ARF and 40 synthetic mesh. While both methods had similar efficacy and reliability, they recommended that ARF be used in patients when synthetic mesh was unavailable, or mesh use was not recommended [17]. El-Gamal et al. studied 44 patients in which ARF was used in mid-section of the sling and branches consisted of synthetic mesh; they concluded that short-term results showed that this method's efficacy and expenses were acceptable [18]. Trabuco et al. conducted a retrospective cohort study on 79 patients who underwent mid-urethral sling operation with ARF and 163 patients in which synthetic mesh was used. They concluded that synthetic mesh was more effective and reliable [19]. Athanasopoulos et al. performed a pubovaginal sling operation with ARF in 264 patients and reported that the sling operation that used ARF had moderate morbidity and was an effective surgical method [20].

Ideal sling material should have low erosion and infective complication rates and should be easily tolerated by the patient [21]. Various synthetic materials have been developed [22]. Many studies have investigated monofilament and multifilament slings made of various materials, and have found that monofilament material with wider gaps has lower complication rates [23].

Along with these studies, it has also been indicated that ARF caused less erosion and infection, and was more easily tolerated due to less tissue reaction [24]. Besides, infectious complications (especially prion) due to sterilization issues of synthetic materials pose a different threat [24,25]. Operations using synthetic materials also have a financial disadvantage due to product costs [26]. However, some studies show that procedures using rectus fascia have longer operation length, blood loss, postoperative pain, and hospitalization length [7,17,27]. These are factors that increase additional morbidity and hospitalization expenses [17,27].

In our study, success rates were consistent with the literature. Our complication rates were low. Also, we did not see complications specific to synthetic material intensely.

Study limitations

This study has some limitations. The number of patients is relatively small and the study was conducted in a single center, making generalization of the results difficult. However, given the absence of a consensus on which treatment method is more effective in urinary stress incontinence, we believe that our findings will be guiding for future studies.

## Conclusions

TOT operation using ARF is just as effective and reliable as synthetic mesh. This choice should be based on effective doctor-patient communication and the patient's clinical history. TOT surgery offers high success rates regardless of the material used. Further studies with broader patient groups should be conducted.
